# What factors contribute to the continued low rates of Indigenous status identification in urban general practice? - A mixed-methods multiple site case study

**DOI:** 10.1186/s12913-017-2017-6

**Published:** 2017-01-31

**Authors:** Heike Schütze, Lisa Jackson Pulver, Mark Harris

**Affiliations:** 1 School of Health and Society, University of Wollongong, Northfields Avenue, Wollongong, 2522 NSW Australia; 2 Office of the Pro-Vice Chancellor - Engagement and Aboriginal & Torres Strait Islander Leadership, University of Western Sydney, Locked Bag 1797, Penrith, 2751 NSW Australia; 3 Centre for Primary Health Care and Equity, Level 3, AGSM Building, UNSW Australia, Sydney, 2052 Australia

**Keywords:** Indigenous status identification, Aboriginal and Torres Strait Islander status, Ethnic monitoring, Aboriginal and Torres Strait Islander health, Primary care, Unannounced standardised patients, Policy

## Abstract

**Background:**

Indigenous peoples experience worse health and die at younger ages than their non-indigenous counterparts. Ethnicity data enables health services to identify inequalities experienced by minority populations and to implement and monitor services specifically targeting them. Despite significant Government intervention, Australia’s Indigenous peoples, the Aboriginal and Torres Strait Islander peoples, continue to be under identified in data sets. We explored the barriers to Indigenous status identification in urban general practice in two areas in Sydney.

**Methods:**

A mixed-methods multiple-site case study was used, set in urban general practice. Data collection included semi-structured interviews and self-complete questionnaires with 31 general practice staff and practitioners, interviews with three Medicare Local staff, and focus groups with the two local Aboriginal and Torres Strait Islander communities in the study areas. These data were combined with clinical record audit data and Aboriginal unannounced standardised patient visits to participating practices to determine the current barriers to Indigenous status identification in urban general practice.

**Results:**

Findings can be broadly grouped into three themes: a lack of practitioner/staff understanding on the need to identify Indigenous status or that a problem with identification exists; suboptimal practice systems to identify and/or record patients’ Indigenous status; and practice environments that do not promote Indigenous status identification.

**Conclusion:**

Aboriginal and Torres Strait Islander peoples remain under-identified in general practice. There is a need to address the lack of practitioner and staff recognition that a problem with Indigenous status identification exists, along with entrenched attitudes and beliefs and limitations to practice software capabilities. Guidelines recommending Indigenous status identification and Aboriginal and Torres Strait Islander-specific Practice Incentive Payments have had limited impact on Indigenous status identification rates. It is likely that policy change mandating Indigenous status identification and recording in general practice will also be required.

## Background

Indigenous populations and other racial minority populations experience poorer health outcomes than their non-indigenous counterparts [1] and receive a lower quality of healthcare [2]. The health disparities between Australia’s Indigenous peoples, the Aboriginal and Torres Strait Islander peoples, and other Australians [3, 4] is the largest observed of all developed countries. Aboriginal and Torres Strait Islander peoples represent approximately 2.5% of the population yet contributed to 3.6% of the total burden of disease [5]. Chronic diseases account for 80% of the difference in the burden of disease observed between Aboriginal and Torres Strait Islander peoples and other Australians [6]. The greater proportion of studies in Aboriginal and Torres Strait Islander health research have been carried out in rural and remote areas and limited information is available in an urban context [7, 8]. The limited data available on the distribution of Aboriginal and Torres Strait Islander morbidity across areas shows that Aboriginal and Torres Strait Islanders are more likely to experience chronic disease at higher rates in the major city areas [9, 10].

Access to primary care has an impact on health outcomes [11] and evidence from Australia, the United States and New Zealand indicates that primary health care can contribute to closing the gap in life expectancy between indigenous and non-indigenous populations [12], presenting an important opportunity to address health disparities.

The collection of ethnicity data supports health services to identify inequalities in health status and health care access, allows targeted population-specific services to be developed, and enables interventions and health outcomes to be monitored [13–15]. Indigenous status needs to be recorded in order to offer targeted services to Aboriginal and Torres Strait Islander patients in general practice. In 2012 just over half (59%) of the total Aboriginal and Torres Strait Islander population had identified their Indigenous status under the Medicare Voluntary Indigenous Identification program [16], resulting in 41% of the Aboriginal and Torres Strait Islander population’s Medicare claims not being counted towards Aboriginal and Torres Strait Islander health statistics. Figures derived from general practitioner (GP) reports show a similar picture, with 1.5% of all encounters in general practice in 2012–2013 recorded as being with Aboriginal and Torres Strait Islander patients [17].

Several barriers to Indigenous status identification in general practice have been identified, including a lack of effective routine identification processes [18–22], software systems that do not allow Indigenous status to be recorded according to National Best Practice Guidelines [18, 19, 23], an assumption that there are no Aboriginal and Torres Strait Islander patients at the practice [18, 23] or that Indigenous status can be ascertained by physical appearance [18, 20].

**Fig. 1:**
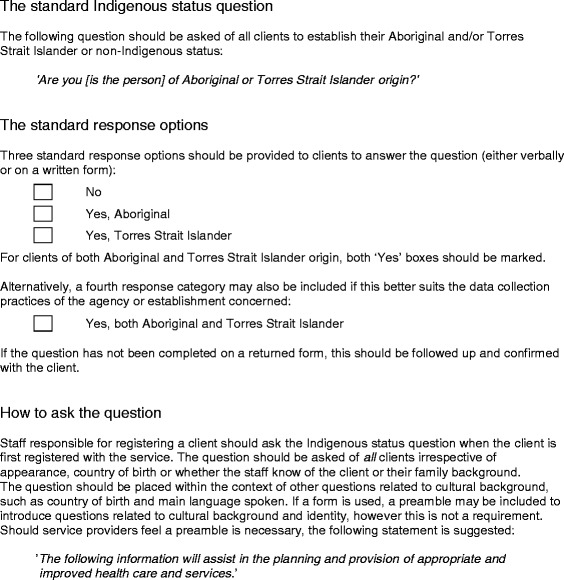
Outline of the National Best Practice Guidelines for Collectiing Indigenous Status in Health Data Sets [24] (p9)

A number of strategies have been put in place to improve Indigenous status identification in Australia. In 2010 the *National Best Practice Guidelines for Collecting Indigenous Status in Health Data Sets* [24] was released, which provided comprehensive guidance on how to collect and record Indigenous status (see Fig. 1 for an outline of the standard question, stating it should be “*asked of all clients irrespective of appearance, country of birth or whether the staff know of the client or their family background”* [24] (p9)). Although the recording of Indigenous status is compulsory in the public sector [25], it is not mandatory in general practice whose processes are guided largely by the industry standards, the *Standards for General Practices* [26], set by the Royal Australian College of General Practitioners (RACGP). The RACGP released a new edition of their guidelines, *Standards for General Practices (4th edition)* [26]*,* which included alignment with the *National Best Practice Guidelines,* and in 2011 they also released *Identification of Aboriginal and Torres Strait Islander People in Australian General Practice* [27]. In addition to these publications, the Australian Government introduced the $805 million *Indigenous Chronic Disease Package* [28] in 2010 in response to the *National Partnership Agreement on Closing the Gap on Indigenous Health Outcomes* [29]. This included funding to employ Aboriginal outreach workers to help with access issues, ‘Closing the Gap’ Officers to increase awareness in general practice, reduced cost prescriptions for eligible Aboriginal and Torres Strait Islander patients, and additional payments to GPs for the chronic disease management of their Aboriginal and Torres Strait Islander patients. Despite the availability of guidelines and additional funding, Aboriginal and Torres Strait Islander patients continued to be under identified in general practice data sets [18, 30], particularly in urban areas [7].

The Australian Institute of Health and Welfare’s 2013 publication, ‘*Taking the next steps: identification of Aboriginal and Torres Strait Islander status in general practice’* [31] states:

*“…only a minority of general practices have effective processes to routinely collect Indigenous status data from patients/clients, and that there are considerable barriers to implementing these processes. In addition, the structure of the general practice sector means that improving Indigenous data collection faces different challenges compared with other health settings”* [31] (p1)….*“Research studies specifically investigating general practice identification processes predate the impact of recent reforms, which have taken place largely since 2010. These studies showed only a minority of mainstream general practices had routine identification processes in place for all patients…While specific investigations have not been repeated since the reforms were implemented, overall data on general practice activity indicate little change in the proportion of patients recorded as Aboriginal or Torres Strait Islander”* [31] (p6).

The Australian Government made a large investment to close the Aboriginal and Torres Strait Islander health gap. Furthermore, 35% of the Aboriginal and Torres Strait Islander population live in major cities [32] and approximately 50% access non-Aboriginal specific primary care (mainstream general practice) at least some of the time [16, 33]. Thus the aim of this study was to explore the current barriers to Indigenous status identification and the provision of appropriately targeted care to Aboriginal and Torres Strait Islander patients in urban general practice two years after the Indigenous health reforms. This study was part of a larger study which aimed to improve Indigenous status identification rates and the acceptability and appropriateness of care provided to Aboriginal and Torres Strait Islander patients in general practice. This paper presents the baseline (pre-intervention) findings on Indigenous status identification. Findings on the provision of targeted care have been previously published [34].

## Methods

### Theoretical underpinning, study design and setting

The main study began with the assumption that the patient experience in general practice was more than just a verbal exchange between patients, staff and providers, but rather that these interactions are complex social processes. This aligned with an interpretivist constructionism view that: “*all human ‘knowledge’ is developed, transmitted and maintained in social situations”* [35] (p15), therefore *“reality is socially constructed”* [35] (p13) through the social processes such as language, engagement and other social interactions.

Case studies allow a detailed, holistic, intensive exploration of individuals, groups, organisations and phenomenon in context [36, 37]. A multiple-site case study allows the researcher to explore differences within and between cases and replicate findings across cases [38–40], and hence was employed for this study as it allowed for the detailed investigation of factors operating both within and across individual general practices. Interviews and focus groups allowed in-depth exploration of beliefs, attitudes and opinions [41], unannounced standardised patients were considered the gold standard for assessing physician performance [42], medical record audits allowed actual recording of Indigenous status to be investigated, and self-report questionnaires were used for data triangulation purposes.

The study was conducted in the Eastern Sydney Medicare Local (ESML) area and South-eastern Sydney Medicare Local (SESML) area. (Medicare Locals were a group of 61 regional organisations across Australia that coordinated healthcare services for a geographic area. They were organised into metropolitan, regional and rural peer groups based on the socioeconomic indexes for areas and remoteness area categories [43]. Medicare Locals have since been reorganised into Primary Health Networks). Based on ABS 2011 census data, the proportion of Aboriginal and Torres Strait Islanders in the SESML area was 0.8% (*n* = 3,816) and 1.3% (*n* = 4,541) in the ESML, although this figure is generally accepted as being a under-estimation [44].

The local meeting place for Aboriginal and Torres Strait Islander peoples in the SESML area was the Kurranulla Aboriginal Corporation. There were two local Aboriginal corporations in the ESML area; the La Perouse/Botany Bay Aboriginal Corporation was run by the direct descendants of the traditional owners and was engaged in this study.

### Recruitment and study population

#### Unannounced Standardised Patients (USP)

Elders from Kurranulla Aboriginal Corporation (SESML area) and the La Perouse/Botany Bay Aboriginal Corporation (ESML area) identified two potential candidates each to be employed as USPs. As Aboriginal and Torres Strait Islanders represented a small proportion of the patient population in many general practices, it was requested that candidates ‘not look obviously Aboriginal’ to decrease the possibility that the USP would be detected. All four candidates were offered employment as a casual research assistant and all four accepted; however, one candidate from the SESML area had to withdraw from training due to personal and health reasons. Two of the USPs were not available at the time of baseline data collection (one from each area) and thus one USP was used for baseline data collection.

##### General practice

The two Medicare Local organisations circulated an expression of interest to all general practices in their area. Each practice was considered a ‘case’ and the unit of analysis. Practices were eligible if a general practitioner (GP) agreed to participate and the practice was not currently engaged in a similar or related study. As this was a pilot study with limited funding, recruitment stopped once three eligible GPs from separate practices in each Medicare Local area were recruited. One GP moved practices and wanted to remain in the study, so an additional practice (case) was recruited in the SESML area, hence a total of four practices (cases) were recruited in the SESML area and three in the ESML area. Given the nature of the intervention and limited funding, recruitment could not be extended to more than one GP within a practice, however, in one large practice two GPs were recruited. Once a GP was recruited, permission to conduct the study was obtained from the practice principal. All administrative and nursing staff from the recruited practices were then invited to participate. In total, 31 out of a possible of 44 participants agreed (eight out of eight GPs, two of four nurses, one of one allied health professional, four of six practice managers and 16 of 25 receptionists).

##### Focus groups

Elders in the local Aboriginal and Torres Strait Islander communities either directly identified potential female participants for the focus groups or nominated a person to act on their behalf. Participants were eligible if they had ever used non-Aboriginal specific general practice. The Elders of the respective communities had communicated to the researcher that discussions surrounding attending general practice were separate ‘women’s’ and ‘men’s’ business and mixed gender focus groups would not be appropriate. Funding was not available to hold separate focus groups with men.

##### Medicare local staff

The Closing the Gap Officers and Practice Support Officers in each Medicare Local area were invited to participate by the researcher. Both Closing the Gap Officers and one Practice Support Officer agreed, the other Practice Support Officer was not available.

### Data collection

Data collection occurred between May and September 2012. All interview and survey guides can be accessed at http://unsworks.unsw.edu.au/fapi/datastream/unsworks:34679/SOURCE02?view=true [45].

#### Unannounced Standardised Patients (USP)

The USP presented covertly as a patient to each practice once (twice to Practice 202 which was the practice that had two GPs recruited) without disclosing that they were the study patient. Immediately after the visit they completed a report which included: whether or not they had been asked their Indigenous status; if literacy was assumed; their perceptions on the practice environment; their level of comfort making the appointment and during the visit; what occurred during the consultation; and whether or not they would return to the practice again if given a choice. The USP was then also interviewed to gather rich data on their experience as a patient which may not have been captured in the self-report survey.

#### Individual interviews

Semi-structured interviews were conducted with 30 of the 31 participating GPs and practice staff; 29 were conducted face-to-face in the participants’ workplace, the other via telephone. Participants were asked 12 broad questions that allowed for flexibility around the responses about their knowledge, attitudes and skills regarding identifying patient’s Indigenous status; the provision of culturally appropriate care to Aboriginal and Torres Strait Islander patients; and their knowledge and attitudes regarding the Aboriginal and Torres Strait Islander-specific Medical Benefits Schedule (MBS) Item Numbers, Pharmaceutical Benefits Scheme (PBS) Co-payment Measure, and Practice Incentive Payments. All interviews were audio recorded with the participants’ consent. The average interview duration was 12 min (range 3–55).

Unstructured face-to-face in-depth interviews were conducted with the two Medicare Local Closing the Gap Officers to gain an understanding of their perceptions and experiences regarding the implementation of, and the effectiveness of Indigenous status identification systems and the existing Aboriginal and Torres Strait Islander-specific health initiatives in general practice. Participants were asked to discuss any current programs to improve culturally appropriate care for Aboriginal and Torres Strait Islander patients and what they felt the barriers and facilitators to these were. Interviews were audio recorded with the participants’ consent. The interviews lasted 1 h 13 min and 1 h 40. To further explore the theme of Practice Accreditation, the Medicare Local Practice Support Officer was interviewed by telephone (22 min 20).

#### Self-complete questionnaires

Self-complete mail questionnaires were administered to the 31 participating GPs and practice staff and 29 were returned. Questionnaires included: demographic questions; the practice’s level of involvement with Aboriginal-specific health services and organisations; the participant’s knowledge and perceptions on the Indigenous status identification methods used in the practice, the barriers and enablers to providing care to Aboriginal and Torres Strait Islander patients, and their views of the Aboriginal and Torres Strait Islander-specific MBS Item Numbers, PBS Co-payment Measure and Practice Incentive Payments; and various aspects of care that had been identified in the literature and focus groups as being important when providing culturally appropriate care to Aboriginal and Torres Strait Islander patients [46, 47]. Some questions were a repeat of those asked in the individual interviews in order to capture as many different views as possible (as it was thought that some participants might have elected to participate only in the interview or the survey, not both), and for data triangulation purposes.

#### Practice summary and systems audit

HS audited each of the seven participating practices to identify what Indigenous status identification systems were in place and to gather information on the practice structure (ownership, accreditation status, number of GPs and staff).

#### Medical records audit

HS manually audited the electronic patient database in each practice to determine the number of patients with Indigenous status recorded, the number of consultations and health checks each Aboriginal and Torres Strait Islander-identified patient had in the previous two years and whether they were enrolled in any Government initiatives such as the Aboriginal and Torres Strait Islander-specific PBS Co-payment Measure. Wherever possible, billing data was crossed-referenced with the medical records in case more Aboriginal and Torres Strait Islander-identified patients could be identified.

#### Focus groups

To gather a broader view of the factors that were deemed important in the patient journey, a focus group was conducted at a local meeting place in each of the two Aboriginal and Torres Strait Islander communities within the Medicare Local study areas, with five participants in one group and six in the other. Focus groups were audio-recorded with the participants’ consent. An Associate Investigator (themselves a member of the local Aboriginal and Torres Strait Islander community) acted as note-taker for each focus group. The aim of holding the focus groups at local community meeting places was to help participants feel more comfortable and to aid open discussion, as was having an ‘insider’ as note-taker [41]. One focus group lasted 1 h 12 min, the other 1 h 22. Participants were given a $30.00 gift card to compensate for their time.

### Data analysis

The interview and focus group recordings were transcribed verbatim. Thematic analysis was performed according to the method described by Braun and Clarke [48] in Nvivo [49] Version 9.2, a program that assists with coding and data organisation. HS developed the initial code frame for the interviews and focus groups; LJP and MH reviewed the coding of five interviews and the focus groups to identify differing or additional insights or meanings, which then informed the subsequent analysis. Data saturation was reached for the interviews and focus groups.

A number of steps were undertaken to improve the rigour of the study. To increase credibility, the coding and analysis of the Medicare Local staff interviews were provided back to the respondents to verify that the interpretations were correct (a process known as member checking or participant validation) [50]. In the case of focus groups, a group representative was provided with the analysis. GPs and practice staff were not asked to validate the interpretations of their interviews as this may have influenced their response to the invention. In addition to this, HS spent sufficient time in the field to understand the culture of the respondents and their setting (prolonged engagement), a USP observed usual behaviour (used instead of prolonged engagement), data was collected from multiple sources (source triangulation), multiple data collection techniques were used (data triangulation), the analysis was performed using multiple researchers (researcher triangulation), and ‘exception to the rule cases’ were carefully examined (deviant case analysis) [50, 51]. Transferability was enhanced by providing sufficient description and context as well as explicitly stating that interpretivist constructionism underpinned the research [50]. Data triangulation and a detailed account of the data collection, analysis methods and the theory underpinning the research were used to increase dependability [52]. Confirmability was achieved by explicitly stating the researcher’s perspective, and through data and researcher triangulation [50, 53].

Simple descriptive statistical analyses were performed on the quantitative data from USP visits, audit data and self-report questionnaires using SPSS Version 21 [54].

## Results

### GP/staff attitudes and beliefs regarding the need to identify Indigenous status

There was a lack of GP and staff awareness and large variability within and between practices as to the reason why Indigenous status was recorded and that a problem existed with the identification of patients’ Indigenous status. A number of staff were not aware there were barriers; others felt there none:

*“What’s the problem with identification, you just look at them and then you know if they’re Aboriginal?”* General Practitioner, Practice #101

It was common for participants to believe that there were no Aboriginal and Torres Strait Islander patients at their practice:

*“We don’t have any Aboriginal patients here…I mean it’s a family practice and so they don’t really come here for their sort of problems…”* General Practitioner, Practice #103

Several believed that patients did not fill in forms correctly or that Aboriginal and Torres Strait Islander patients were reluctant to identify:

*“They don’t want to identify uh for whatever reason because, because I guess they’re a minority and they want to be like everybody else.”* General Practitioner No. 1, Practice #202

It was not uncommon among reception staff to believe that patients would be offended if asked their Indigenous status. Some GPs also believed patients would be offended and therefore only asked patients their Indigenous status if they ‘appeared Aboriginal’:

*“…And some patients are uncomfortable if you ask the question. So sometimes if somebody has got a darkish skin and you ask them are you from an Aboriginal background they get upset if they’re not…So the people that’s not ATSI or uh what’s-the-name, so they feel offended, ‘Why did the GP ask me, do I look Aboriginal?’”* General Practitioner, Practice #203

Some mentioned that it was often difficult to identify Aboriginal and Torres Strait Islander patients because their physical appearance was variable:

*“….if they’re only a quarter or an eighth or something Aboriginal, then say skin colour, then you wouldn’t know that they’re Aboriginal…I would say that would be the only barrier.”* Receptionist No. 1, Practice #203

Others felt that it was the patient’s responsibility to self-identify, and that the practice’s responsibility regarding Indigenous status identification ended with providing a question on a new patient registration form:

*“We give everyone a form. We ask people to identify….it’s up to them to decide.”* Practice Manager, Practice #202

### GP/Staff lack of awareness regarding the healthcare needs of Aboriginal and Torres Strait Islander patients

Few participants were aware of the health discrepancies and different health care needs between Aboriginal and Torres Strait Islander peoples and other Australians. Six respondents (20%) indicated they had undertaken some Aboriginal and Torres Strait Islander Cultural Awareness Training previously. No practice had any engagement with an Aboriginal Medical Service (AMS) or Aboriginal Community Controlled Health Service (ACCHS); one practice had some engagement with a local Aboriginal organisation.

### Practice Indigenous status identification systems

#### Staff knowledge of their Indigenous identification systems

Table 1 shows that most participants stated that there was a question on the *New Patient Registration Form* to identify the Indigenous status of new patients; 25% GPs and 19% staff did not know how Indigenous status was identified for new patients. With regards to existing patients, 25% GPs and 48% staff stated that they identified the Indigenous status of existing patients via a form, however, only one practice had a form for existing patients. Twenty-five percent GPs and 14% staff did not know that Indigenous status was recorded in the medical record.

**Table 1:** GP and staff awareness of their Indigenous status identification systems

Indigenous status identification systems	General practitioner (*n* = 8)	Nurse (*n* = 2)	Practice manager (*n* = 4)	Receptionist (*n* = 15)
Not aware how Indigenous status identified for new patients	2	0	0	4
Not aware how Indigenous status identified for existing patients	2	0	1	2
Indigenous status not asked for existing patients	1	0	0	3
Not aware Indigenous status recorded on the medical record	2	0	1	2

Within each practice, there was a variety of responses as to whether reception, GPs or nurses checked the Indigenous status of both new and existing patients, with some saying that reception followed up, some saying GPs followed up, some saying both, some saying neither.

#### Indigenous status identification and recording processes

Most practices (six) had an Indigenous status question on their *New Patient Registration Form*. No practices asked new patients their Indigenous status according to the *National Best Practice Guidelines for Collecting Indigenous Status in Health Data Sets* [24] (see Fig. 1 for how the question should be worded). One practice had a system in place to identify the Indigenous status of existing patients and this was done according to the *National Best Practice Guidelines* and offered patients an explanation of why they were being asked the question.

Table 2 shows that four of seven practices did not have Indigenous status recorded for 86–100% of patients. Practice 101 had the Indigenous status recorded for 100% of patients; 59% were recorded as ‘Refused/Inadequately Stated’. Further investigation revealed that the practice had guessed the Indigenous status of many patients and coded the remaining patients as ‘Refused/Inadequately stated’. The Indigenous status was not recorded for 26.2% of patients for Practice 201. The researcher was informed that they were progressively working through their records “alphabetically” to update the Indigenous status of their patients. It appeared to the researcher that staff were working through their patient records alphabetically and guessing patients’ Indigenous status.

**Table 2:** Indigenous status of patients ≥18 years (practices *n* = 7)

Practice code	Indigenous status (%)			
	Indigenous	Non- Indigenous	Refused/Inadequately stated	Unidentified
101	0	41	59	0
102	0	14	0	86
103	0	0	-	100
104	0.1	4	-	96
201	0.1	73.2	-	25.7
202	0.2	54.3	-	45.5
203	1.3	0	-	98.7^a^

- Practice software does not have refused/Inadequately stated option

^a^Some patients had an ethnicity other than Aboriginal and/or Torres Strait Islander recorded but the practice software did not record these patients as being non-Indigenous

The above findings were supported by the USP visits. The USP was instructed that when they presented to a practice, if there was an Indigenous status question on the registration form to leave it blank to see if staff would prompt them to answer the question and whether it would be done according to the *National Best Practice Guidelines*. Table 3 shows that most practices relied solely on patients to self-identify their Indigenous status on a *New Patient Registration Form*. Less than half of the practices prompted the USP to complete the Indigenous status question if left unanswered; the USP’s Indigenous status was correctly recorded only half of the time, and two separate practices recorded the USP as non-Indigenous based on physical appearance alone. Results appear similar regardless of whether practices were accredited, non-accredited, solo-GP, multi-GP, practitioner-owned or corporation-owned.

**Table 3:** How the USPs Indigenous status was identified and recorded (eight USP visits to seven practices)

	USP visits (*n* = 8)	
	Yes	No
USP asked their Indigenous status	7 *	1
* Yes - asked on registration form	6	2
* Yes - asked by reception	3	5
* Yes - asked by Physician	2	6
Indigenous status asked according to Best Practice Guidelines	0	8
USP Indigenous status correctly recorded in medical record	4	4

### Practice environments

One practice had a large piece of Aboriginal artwork at reception. Apart from this, no practice had any posters, signs, brochures or other health information that either mentioned or depicted Aboriginal or Torres Strait Islander people or encouraged patient’s to disclose their Indigenous status.

### Focus group participant views

Focus group participants identified visual symbols of welcome such as the Aboriginal and Torres Strait Islander flags and artwork or signage as important factors in the patient journey, stating that these showed that Aboriginal and Torres Strait Islanders were welcome. Staff attitudes and behaviours played an important role in making patients feel welcome and a patient’s experience in the waiting room and at reception was just as important as the consultation. Barriers included appointment wait times, feeling rushed in the consultation, and not having information explained in a way that could be understood. Some participants were not aware of why their Indigenous status was collected, whilst others believed it was for census purposes or was linked to their social security payments. Focus group participants in both communities said that they would not be offended being asked their Indigenous status if it were asked in an appropriate manner and an explanation for why the question was being asked was also provided.

## Discussion

This study demonstrated that the Indigenous status of patients was not being adequately identified and recorded in seven urban general practices in two Medicare Local areas in Sydney. This suggests that there has been little change to Indigenous status identification since the introduction of the Indigenous health reforms in 2010. Most practices were relying solely on patients to self-identify their Indigenous status on a *New Patient Registration Form* and they did not have established systems in place to identify the Indigenous status of existing patients. Only one practice asked patients their Indigenous status according to the *National Best Practice Guidelines* (although this was only for existing patients, not new patients). Participants in over half the practices externalised the problem of Indigenous status identification to the patient, with GPs and/or staff stating that they did not have any Aboriginal and Torres Strait Islander patients, patients did not complete forms correctly, or patients would be offended if asked their Indigenous status, and several participants believed patients Indigenous status could be determined by physical appearance. Very few GPs and practice staff internalised the issue as being due to their own practice routines and systems, or their assumptions and attitudes regarding where Aboriginal and Torres Strait Islander patients sought health care and for what reasons. When the USP presented at each practice, she was asked to self-identify her Indigenous status on a *New Patient Registration Form* at five practices; only three practices prompted the USP to complete the field when it was left blank, and the USP’s Indigenous status was incorrectly recorded as non-Indigenous in two practices based on physical appearance.

Previous studies investigating Indigenous status identification methods in general practice have found that one third of GPs reported that they did not routinely ask patients their Indigenous status, whilst two-thirds reported that they assumed Indigenous status based on local knowledge or physical appearance, and only asked patients if they thought they were of Aboriginal and Torres Strait Islander descent, or relied on the patient to self-identify [20, 21]. When recruited into the current study, most GPs stated that they routinely asked all patients their Indigenous status. Although a there was a much smaller sample size in the current study, social desirability bias could explain the difference, as the two previous studies were conducted in 2003 and 2004 prior to any guidelines for Indigenous status identification in general practice in Australia being released [55].

A strength of the current study is that it did not solely rely on self-reported data and triangulated data which helped to overcome the inherent biases of using one data source alone [50, 51]. Self-reported responses were checked against patient records, each practice’s Indigenous status identification systems were audited, and a USP tested the actual Indigenous status identification processes within each practice. The results demonstrate that although many GPs and staff believed they routinely identified patients’ Indigenous status, they did not. These findings are supported by a study in the Australian Capital Territory (ACT) where only six of 28 Aboriginal and Torres Strait Islander people interviewed reported that they had been asked their Indigenous status (although it is not clear how many of the six were asked and how many volunteered the information without being prompted either verbally or via a question on a form) [22].

A national study of Indigenous status identification methods in general practice found a common barrier was an assumption that the practice did not have any Aboriginal and Torres Strait Islander patients because Aboriginal and Torres Strait Islander patients only used Aboriginal-specific health services [18]. Few participants in the current study were aware of having any Aboriginal and Torres Strait Islander patients and most believed that they did not have any. This lack of awareness resulted in several GPs and/or practice staff believing it was not necessary to ask patients their Indigenous status because it was unlikely that they would see an Aboriginal or Torres Strait Islander patient (a view echoed by GPs in studies in the ACT [23] and Queensland [21]). Two GPs stated that theirs was a family practice and therefore not utilised by Aboriginal and Torres Strait Islander patients and hence they did not need to ask Indigenous status. This subtle yet pervasive institutional and interpersonal racism (whether subconscious or willing) will need to be addressed at the national level as the effects of racism on the health of indigenous populations is well recognised both nationally [56, 57] and internationally [58, 59]. Some participants believed that they did not need to ask Indigenous status because patients’ Indigenous status could be determined by physical appearance, which has also has been found in two previous studies, with participants stating they only needed to ask the question of people who ‘appeared Indigenous’ [18, 20].

Similar to findings in other studies [18, 21, 23], some GPs and practice staff in this study were not comfortable asking patients their Indigenous status due to concerns about offending non-Indigenous patients or because they felt it was discriminatory to ask patients their Indigenous status. This discomfort appeared to be based on a lack of understanding of why Indigenous status was collected and because staff did not have an adequate response for patients when they queried why they were being asked.

A common finding across the current and previous studies was a view that it was the patients responsibility to identify their Indigenous status [18, 20]. Similar to what has been reported elsewhere [20], several participants assumed that Aboriginal and Torres Strait Islander patients were reluctant to identify their ethnicity. The Aboriginal and Torres Strait Islander focus group participants in the current study stated that they would not be offended being asked their Indigenous status if it were asked in an appropriate manner and an explanation for why the question was being asked was also provided; this has also been found in the ACT [22] and Queensland [20]. Although it does not specifically pertain to the general practice setting, a national study by the Australian Bureau of Statistics on Aboriginal and Torres Strait Islander peoples perspectives’ on having Indigenous status recorded [60] also supports these findings.

Another factor contributing to the low levels of Indigenous status identification in general practice is that the recording of Indigenous status is not mandated in general practice. General practice is largely under private ownership and self-regulation against the *Standards for General Practice* [26] set by the Royal College of General Practitioners (RACGP) is optional. Additionally, the RACGP Standards prior to 2010 were ambiguous and the importance of collecting Indigenous status was blurred with the general collection of ethnicity data on other high need groups [55, 61]. In 2010, the RACGP released an updated version of their Standards [26] which aligned with the *National Best Practice Guidelines.* However, to allow for transition time between versions, the recommendations in both editions of the RACGP Standards were considered current up until 30 October 2014. Although having one uniform set of Standards may improve understanding of the recommendations for Indigenous status identification in general practice, this may not have the same impact as a nation-wide policy mandating that Indigenous status be collected. This might be achieved by requiring that Indigenous status be recorded on a Medicare claim in order for GPs to receive payment. However, careful measures would need to be put in place to ensure that Indigenous status was accurately collected and recorded. Incentives to increase GP and staff awareness of the health needs of Aboriginal and Torres Strait Islander people could also help address this.

The ability to record Indigenous status in electronic patient records in general practice software was also a barrier to identification processes [18, 19] and continues to be so. Multiple software packages are available to handle patient records and no standards have been mandated [62], resulting in some software packages not being able to record Indigenous status, some packages have a ‘refused’ option to enable staff to ascertain whether or not the patient has already been asked their Indigenous status, whilst the default in some packages automatically records patients as non-Indigenous as opposed to leaving the Indigenous status field unanswered [18, 19, 23].

This study supports the findings of previous studies [18, 20, 21, 23, 63] and adds to the literature by demonstrating that patients are not routinely being asked their Indigenous status in some urban general practices in two areas of Sydney. It demonstrates that misconceptions regarding Indigenous status identification in general practice persist to date and provides further evidence that Aboriginal and Torres Strait Islander people are happy to be asked their Indigenous status if it is done in an appropriate manner. It demonstrates that in order to address the low rates of Indigenous status identification in general practice, the fundamental issues of the lack of awareness that a problem exists with Indigenous status identification must be addressed, as well as entrenched attitudes and beliefs, and practice software capabilities. It further adds to the literature by demonstrating that guidelines recommending Indigenous status identification and Aboriginal and Torres Strait Islander-specific Practice Incentive Payments appear to have had limited impact on Indigenous status identification and suggests that policy change mandating Indigenous status identification and recording in general practice will also be required.

### Study limitations

Participants self-elected to be involved in the research and may represent a group of motivated practitioners and practice staff, however, the characteristics of providers are broadly similar to those in general practice in Australia [62]. The results indicated a lack of knowledge, awareness and practice systems consistent with the findings of previous research [18, 20–23], indicating that the sample was not positively biased.

Participating GPs were from two urban areas in Sydney and may not be representative of GPs in all urban areas. As a low number of practices were involved in the study, the results may not be transferable to other settings. However, the participating practices included a mix of solo- and multi-GP practices, practitioner- and corporation-owned practices, and the majority were accredited practices, representing a good sample of practices.

The focus groups were conducted with women only as the communities communicated that discussions surrounding medical appointments were not appropriate with mixed gender groups. Having same sex participants may have allowed for more free and open conversation among the participants [41, 64], increasing the likelihood that all relevant information was obtained in the focus groups. It is possible, however, that men may have identified others factors as being important to them which women did not. Limited funding did not permit an additional researcher to conduct focus groups with men.

The USP visits were carried out by a single USP visit to six practices and two visits to separate GPs at a seventh practice. The service provided by the GPs and staff on the day of the USP visit may reflect their actions on a particular day and not in general. The use of USPs can be strengthened by using multiple USPs over a number of visits to each practice to reduce the likelihood of biased assessments [65]. Multiple USP assessments were not possible in this study due to limited funding. The USP used was female and her views on the acceptability of health care could differ from males, and the use of both male and female USPs may have strengthened the study.

## Conclusion

Aboriginal and Torres Strait Islander peoples are under identified in general practice. There is a need to address the lack GP and practice staff recognition that a problem with Indigenous status identification exists, along with entrenched attitudes and beliefs and limitations to practice software capabilities. Guidelines recommending Indigenous status identification and Aboriginal and Torres Strait Islander-specific Practice Incentive Payments appear to have had limited impact on Indigenous status identification. It is likely that policy change mandating Indigenous status identification and recording in general practice will also be required.

## Abbreviations

ACCHS: Aboriginal Community Controlled Health Service
AMS: Aboriginal Medical Service
GP: General practitioner
IHIPIP: Indigenous Health Incentive Practice Incentive Program
MBS: Medical benefits schedule
PBS: Pharmaceutical Benefits Scheme
PIP: Practice incentives program
RACGP: Royal Australian College of General Practitioners
USP: Unannounced standardised patient
